# Get a New Perspective on EEG: Convolutional Neural Network Encoders for Parametric t-SNE

**DOI:** 10.3390/brainsci13030453

**Published:** 2023-03-07

**Authors:** Mats Svantesson, Håkan Olausson, Anders Eklund, Magnus Thordstein

**Affiliations:** 1 Department of Clinical Neurophysiology, University Hospital of Linköping, 58185 Linköping, Sweden; 2 Center for Social and Affective Neuroscience, Linköping University, 58183 Linköping, Sweden; 3 Center for Medical Image Science and Visualization, Linköping University, 58183 Linköping, Sweden; 4 Department of Biomedical and Clinical Sciences, Linköping University, 58183 Linköping, Sweden; 5 Department of Biomedical Engineering, Linköping University, 58183 Linköping, Sweden; 6 Division of Statistics & Machine Learning, Department of Computer and Information Science, Linköping University, 58183 Linköping, Sweden

**Keywords:** EEG, deep learning, convolutional neural networks, t-SNE, categories

## Abstract

t-distributed stochastic neighbor embedding (t-SNE) is a method for reducing high-dimensional data to a low-dimensional representation, and is mostly used for visualizing data. In parametric t-SNE, a neural network learns to reproduce this mapping. When used for EEG analysis, the data are usually first transformed into a set of features, but it is not known which features are optimal. The principle of t-SNE was used to train convolutional neural network (CNN) encoders to learn to produce both a high- and a low-dimensional representation, eliminating the need for feature engineering. To evaluate the method, the Temple University EEG Corpus was used to create three datasets with distinct EEG characters: (1) wakefulness and sleep; (2) interictal epileptiform discharges; and (3) seizure activity. The CNN encoders produced low-dimensional representations of the datasets with a structure that conformed well to the EEG characters and generalized to new data. Compared to parametric t-SNE for either a short-time Fourier transform or wavelet representation of the datasets, the developed CNN encoders performed equally well in separating categories, as assessed by support vector machines. The CNN encoders generally produced a higher degree of clustering, both visually and in the number of clusters detected by k-means clustering. The developed principle is promising and could be further developed to create general tools for exploring relations in EEG data.

## 1. Introduction

**Figure 1:**
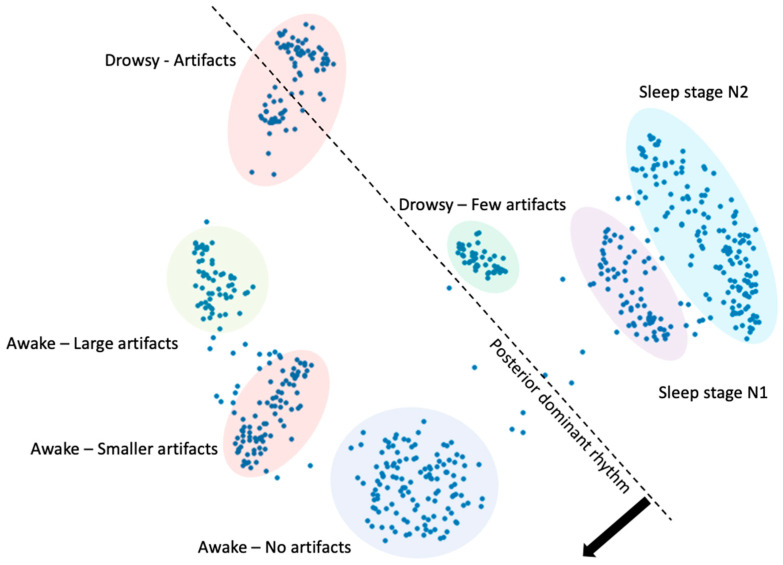
Low-dimensional representation of a 21-channel EEG with a 22 min duration, where each dot represents 2 s of EEG. The figure was generated by a convolutional neural network trained using the principle described in this article, which is based on t-SNE. With a sampling frequency of 250 Hz for the raw EEG, this represents a reduction from 6,930,000 to 1320 data values. A comparison with the original EEG was conducted, and based on this, the structure of the low-dimensional representation is indicated in the figure. The comparison was carried out by visually inspecting the EEG segments corresponding to the dots and characterizing each cluster. The dashed line indicates a boundary, where a posterior-dominant rhythm was present in EEG segments corresponding to the dots to the left of the line.

To understand data, it is necessary to identify important features and, from this, construct categorizations representing dimensionality reductions. For electroencephalography (EEG), the categorization has, historically, mainly depended on human experts. There are several problems with this: (1) it is not known if the categories are the most optimal; (2) it is difficult to provide objective definitions of the categories; and (3) the expert assessments may suffer from intra- and inter-rater variability. When developing algorithms or machine learning to analyze EEG, the data often need to be transformed into a set of features, e.g., a limited set of numbers related to the frequency content. However, important information may be lost in this transformation. In the context of supervised learning, using a ground truth created by experts transfers the inherent problem of the categorization to the method. Hence, methods that discover relevant EEG features and how these are related through a self-supervised process may provide a new perspective on traditional EEG categories and possibly new insights. To this end, in the work presented here, a deep learning approach for improving parametric t-distributed stochastic neighbor embedding (t-SNE) for visual EEG analysis was applied. This article has been written with both clinical neurophysiologists and data scientists in mind. Many of the technical details have therefore been placed in appendices.

t-SNE is a method primarily developed for visualizing high-dimensional data by mapping them to a low-dimensional space [1]. The result is usually clustering of similar data in the low-dimensional representation, and relations in the data can then be identified by visual inspection and comparisons with the original data (Figure 1).

t-SNE is based on pairwise matching of the probabilities that data examples are neighbors in both the high- and the low-dimensional space. The high-dimensional probability has a multivariate normal distribution, and the low-dimensional probability has a multivariate t-distribution. Optimization is accomplished via gradient descent. The original implementation does not create a model for the mapping from high- to low-dimensional representations (the method is described in more detail in Appendix B.1). The original t-SNE is used for the visualization of the results in several EEG studies [2,3,4,5,6,7,8,9,10,11]. In a couple of other studies, the method is used for feature extraction from EEG [12,13].

**Figure 2:**
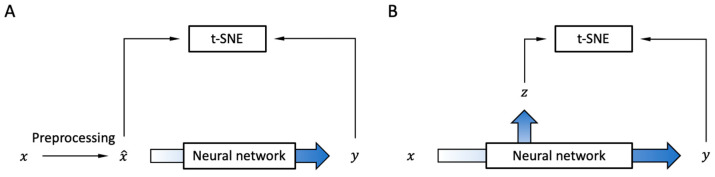
Comparison of the original parametric t-SNE and the suggested method of this work. Both methods produce a low-dimensional representation y of the high-dimensional data x using a neural network. (**A**) The original parametric t-SNE. The input is usually a preprocessed version $\hat{x}$ of the EEG. The optimal way to preprocess the EEG is not known. (**B**) The suggested method. The input x is the raw EEG data, and a new high-dimensional representation z of the data is extracted from the latent space of the network. An advantage of this approach is that the preprocessing step necessary in (**A**) is eliminated.

Parametric t-SNE is a variation where the dimensional reduction is learned by a neural network [14], thus creating a model for the mapping (Figure 2A). In the original implementation, restricted Boltzmann machines are pretrained using greedy layer-wise training and then fine-tuned using the principle of t-SNE. Li et al. [15] and Xu et al. [16] used parametric t-SNE as a step to extract features of motor imagery EEG data, and in the evaluation with support vector machine classifiers, it compared favorably to other feature extraction methods.

Substantial preprocessing is usually required to extract features from EEG. Jing et al. used frequency features in combination with several other statistical or nonlinear measures [2]. Suetani and Kitajo employed frequency features in a modified version of t-SNE using a beta-divergence as a distance measure [6]. Kottlarz et al. used t-SNE to compare frequency features and features created using ordinal pattern analysis [9]. Li et al. used wavelet-generated features for parametric t-SNE [15], and Xu et al. extended this by combining wavelet features with the original EEG and frequency band features, all processed further through common spatial pattern filtering [16]. Other researchers created features using tensor decomposition [3], connectivity measures [4], and several extract features from the latent spaces of neural networks [5,7,8,10]. Exceptions to the above are Ma et al. [12], Georg et al. [11], and Yu et al. [13], who applied t-SNE to raw EEG to create features. It is difficult to assess how the different approaches compare in terms of performance, and none of them can be regarded as a standard approach.

A high-dimensional representation similar to how an expert views EEG might be preferable. Here, the identification of high-level features, such as interictal epileptiform discharges (IEDs), is regarded as important, whereas their time and location may be less important. Using the Euclidean distance of the raw data to compare visually similar examples could result in them being assessed as different, if important waveforms are located at different positions. Furthermore, if the waveforms only constitute a small part of the signals, they may not have a large enough impact on the measure. A property often claimed, regarding convolutional neural networks (CNNs), is the location invariant detection of features [17]. Therefore, using a CNN with appropriate fields of view to convert the EEG into a set of high-level features appears to be a possible solution, but the question of how to train the network needs to be addressed. Training the networks in classification tasks may be an alternative [5,7,10], but this may put too much emphasis on features specific to the classification task and dataset, and involves supervised learning. Deep clustering is a set of methods with which clustering is performed on latent representations in neural networks. Most of the work has been conducted in image analysis, and the methods have been thoroughly reviewed [18,19,20,21]. Many of the methods have similarities to t-SNE, mainly regarding the use of the Kullback–Leibler divergence to match distributions. However, in comparison to t-SNE, there are some notable differences: (1) the number of clusters must often be predefined, sometimes with cluster centers initiated by, e.g., k-means clustering; (2) there is variation in which distributions are used; (3) the loss is often a combination of the Kullback–Leibler divergence and some other form of loss, e.g., the reconstruction loss of an autoencoder; and (4) the network may be pretrained, e.g., as an autoencoder or generative adversarial network.

Currently used EEG categories, e.g., epileptiform discharges and seizure activity, are clinically useful concepts, but the categories are difficult to define objectively, and in praxis, the assessment may differ from expert to expert. t-SNE has been used to visualize EEG in several studies. Motivated by the problem inherent in EEG categorization, the objective of this work was to further improve parametric t-SNE as a tool for the visual study of categories in EEG data. Previous implementations of t-SNE use the raw or, most often, preprocessed EEG as the high-dimensional representation (Figure 2A). Here, CNNs were instead trained using the principle of t-SNE to match a neighbor structure in its latent space to one in its output space (Figure 2B), i.e., the CNNs learned a new relevant high-dimensional representation. In addition, the computations were simplified by using a simple distribution based on ranked distances instead of a normal distribution for the high-dimensional representation. This reduced training times. The main advantage of the suggested method is that no feature engineering is necessary. This may avoid problems with the loss of relevant information due to the choice of an inferior preprocessing technique. The method is fully self-supervised and thus avoids potential biases regarding the definitions of categories introduced in supervised alternatives.

## 2. Materials and Methods

### 2.1. EEG Data

#### 2.1.1. TUH EEG Corpus

All EEG data used in this study were retrieved from the large public database of the Temple University Hospital, Philadelphia—the TUH EEG Corpus [22]. The database contains a mixture of normal and pathological EEGs, and the raw data are unfiltered. The recording electrodes are positioned according to the international 10–20 system [23]. The TUH EEG Corpus (v1.1.0) with average reference was used (previously downloaded in the context of other projects from 17–21 January 2019).

#### 2.1.2. Ethical Considerations

The TUH EEG Corpus was created following the HIPAA Privacy Rule [22]. No personal subject information is available, and there is no way of tracing the data back to the subjects. It was therefore not deemed necessary to seek further approval for this study.

#### 2.1.3. Extracted Data

From the TUH EEG Corpus, three datasets of examples with a 1 s duration were created: (1) wakefulness and sleep (24,647 examples of wakefulness from 19 subjects and 14,450 examples of sleep from 14 subjects); (2) IEDs (4384 examples containing IEDs and 7944 examples without IEDs from 31 subjects); and (3) seizure activity (2549 examples of seizure activity and 7467 examples without seizure activity from 6 subjects). The data extraction and annotation processes are described in more detail in Appendix A. Each dataset was split into approximately 75% for training and 25% for testing. For dataset 1, the test data consisted of new subjects. There were large inter-subject differences regarding the waveform morphology of the IEDs and the character of the seizures. Given the relatively small number of subjects, it was assumed that generalization would be inferior compared to the sleep data. For datasets 2 and 3, the test data therefore consisted of new examples from the same subjects that were used for training. The standard electrodes of the international 10–20-system were used: Fp1, F7, T3, T5, Fp2, F8, T4, T6, F3, C3, P3, O1, F4, C4, P4, O2, Fz, Cz, Pz, A1, and A2 (this is also the channel order used in the analyses). Average reference was used.

#### 2.1.4. Preprocessing

The data were band-pass-filtered between 1 and 40 Hz via Butterworth zero-phase filtering. The data were normalized to ensure the values mostly varied within the interval −1 to 1 by dividing them by the 99th percentile of the absolute amplitude of the training data.

### 2.2. Encoders

Details regarding the network architecture, hyperparameters, and training of the encoders are presented in Appendix C.

#### 2.2.1. Modification of t-SNE

As described in the Introduction and Appendix B.1, t-SNE is based on matching probabilities of data examples being neighbors in a high- and a low-dimensional representation of the data. The suggested approach uses a high-dimensional representation of the EEG data that is generated by the CNN encoders. This means that the representations change during training, and it is necessary to generate them for every batch of training data to compute the corresponding probability distribution. To simplify the computations, a simple distribution based on ranked distances was used instead of a normal distribution. To compute the former distribution, the distances between examples were ranked in increasing order, and the n closest relatives to each example were regarded as neighbors, and therefore given a high probability of being neighbors, while all others were given a low probability. The parameter n thus has a similar function to perplexity in the original t-SNE (see Appendix B.2 for a more detailed description).

#### 2.2.2. CNN Encoder Architecture

**Figure 3:**
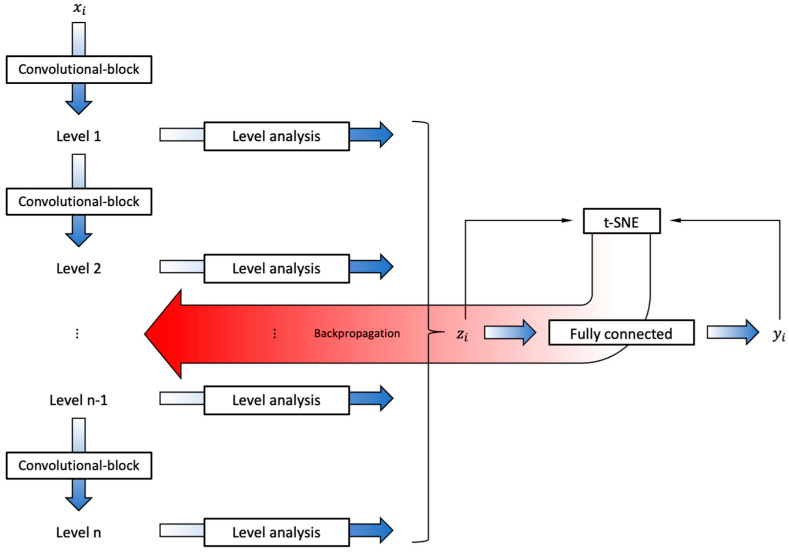
Illustration of the CNN encoder architecture. To the far left, a series of blocks of convolutional and max-pooling layers generate levels with an increasing field of view of the EEG ($x_{i}$). Each level is then processed by further convolution, max-pooling, and a small fully connected layer to produce a set of values which together form the latent representation $z_{i}$ of $x_{i}$. A fully connected network then maps $z_{i}$ to a low-dimensional representation $y_{i}$. The red arrow indicates the backpropagation to adjust the trainable parameters of all layers, where the principle of t-SNE is used to compute the loss.

The CNN encoders consisted of two parts: one part that learned to produce a high-dimensional representation $z_{i}$, and another part that, in turn, mapped this to a low-dimensional representation $y_{i}$ (Figure 3).

The first part had a structure that created levels with different fields of view of the data, and then extracted values that reflected the character of each level. The values together then formed the high-dimensional representation of the data. This was implemented as a series of convolutional and max-pooling blocks, with the latter also down-sampling the data, thereby producing a set of levels. The levels were then processed by a combination of convolutional, max-pooling, and small fully connected layers, and the resulting features from all levels were concatenated to form the high-dimensional representation. EEG channels were analyzed separately in the first part.

The second part was implemented as a series of fully connected layers, where the last layer had two nodes that produced the low-dimensional representation.

A summary of the main encoder configurations is presented in Table 1. For further information on the architecture, see Appendix C.

### 2.3. Evaluation

CNN encoders were trained for the three datasets. Training was performed using a batch size of 500, and 5 neighbors per batch (Appendix B.4).

#### 2.3.1. Comparative Methods

Comparisons of the developed method were carried out with in-house implementations of parametric t-SNE using preprocessed EEG. As accounted for in the introduction, several different techniques have been used for preprocessing EEG for t-SNE, none of them has emerged as a standard technique, and reviewing them in detail is beyond the scope of this work. There are only a few studies using parametric t-SNE for EEG. Li et al. [15] and Xu et al. [16] used Daubechies wavelets [24] for parametric t-SNE. However, in our tests, these did not produce useful results. This was probably due to their discrete nature in conjunction with data examples of a short duration. A continuous wavelet transform (CWT), the Ricker wavelet [25], was therefore tested and found to produce satisfactory results. Xu et al. [16] also used a frequency-band representation of EEG (five bands from the interval 8–30 Hz). As an additional alternative for the comparison, a band representation was therefore created. Compared to Xu et al. [16], higher-frequency resolution and the inclusion of lower frequencies were necessary for useful results, and the short-time Fourier transform (STFT) was chosen as a simple way of implementing this. These implementations are described in more detail in Appendix D. In short, the STFT approach produces a set of values corresponding to frequency bands for short time intervals—in this case, 1 s and 30 bands in the range ~1–30 Hz. This resulted in 630 values per example. CWTs also produce values corresponding to frequency bands for each EEG channel. A total of 29 bands with most of the frequency content below 30 Hz were used, and the following statistics for each band were calculated: mean, standard deviation, minimum, and maximum. This resulted in 2436 values per example. Training was performed using a batch size of 500, and a perplexity of 5, for both feature alternatives. A summary of the main encoder configurations is presented in Table 1.

#### 2.3.2. Quantitative Measures

The developed method and parametric t-SNE were here used to produce two-dimensional representations of the EEG data. Quantifying the quality of an image appears difficult, but two types of measures that reflect how the categories are separated were produced in addition to the resulting images by means of support vector machines (SVMs) and k-means clustering.

The three datasets presented in Section 2.1.3 each consisted of two categories. Using 0 and 1 as labels for the categories of each dataset, SVMs were fitted to the resulting low-dimensional representations of the training data and evaluated on both the training and test data to produce quantitative measures of the separability of the categories and generalization of the models. Linear separability would indicate that the global structure is simple. Since t-SNE produces a nonlinear projection of the data with an element of chance, linear separability may not be good even though the categories are well separated in distinct clusters. Separate SVMs were therefore fitted to the data using either a linear or a radial-basis-function (RBF) kernel. The number of examples was different for each category, and a kappa score κ [26] was used instead of accuracy to adjust for chance results. The resulting classifications were also compared using a chi-square test. If there was a significant difference, post hoc analysis was performed comparing the best classification with the two others individually and applying Bonferroni-adjusted *p*-values.

K-means clustering was used to produce a measure of the ability to generate distinct clusters of the data by each method. Tests from 2 to 50 clusters were performed, and the number of clusters returning the best silhouette score [27] was taken as the measure C.

For all measures, higher values were regarded as better. P-values below 0.05 were considered to be significant.

### 2.4. Software and Hardware

The CNN encoders were developed in Python (version 3.7.13) using the API Keras (version 2.8.0) and the programming module TensorFlow (version 2.8.0). The Python library ‘pyEDFlib’ (version 0.1.22) [28] was used to extract EEG data. Filters were implemented using the ‘scipy.signal’ library [29]. Scikit-learn [30] was used for the support vector machines and k-means clustering.

Four different computers (running Ubuntu) were used, equipped with 64–128 GB of RAM, and either one or two of the following Nvidia graphics cards: Quadro P5000, Quadro P5000 RTX, Quadro P6000, Titan RTX, or Titan X.

## 3. Results

### 3.1. Sleep–Wake

All the methods produced high kappa scores (Table 2), but overall, the CWT encoder produced the highest scores, where the scores were significantly higher when using a linear kernel. When training with an RBF kernel, the CNN encoder scored the highest, but for the test data, the STFT and CWT encoders scored equally, with significantly higher scores than the CNN encoder.

**Figure 4:**
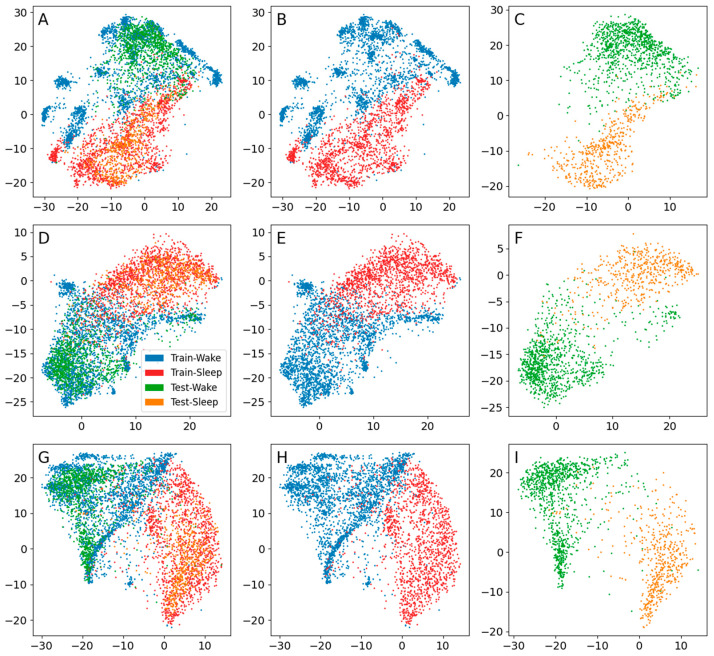
Low-dimensional representations of the sleep–wake dataset. Each dot represents 1 s of data. First row (**A**–**C**): CNN. Second row (**D**–**F**): STFT. Third row (**G**–**I**): CWT. First column (**A**,**D**,**G**): all data. Second column (**B**,**E**,**H**): training data. Third column (**C**,**F**,**I**): test data.

The CNN encoder produced the highest clustering score for the training data (Table 2), where, visually, there was an apparent difference compared to the other methods (Figure 4B,E,H), but for the test data, there were few clusters (Figure 4C), so it generalized but with less substructure. For all methods, the global structure was simple, with two main areas containing most of the respective categories. This was in line with the relatively high kappa scores when using a linear kernel.

### 3.2. IEDs

The CNN encoder produced the highest kappa scores (Table 3), which were significantly higher for all but the test data when using an RBF kernel. The CWT encoder scored higher than the STFT encoder, where the latter scored 0 when using a linear kernel.

**Figure 5:**
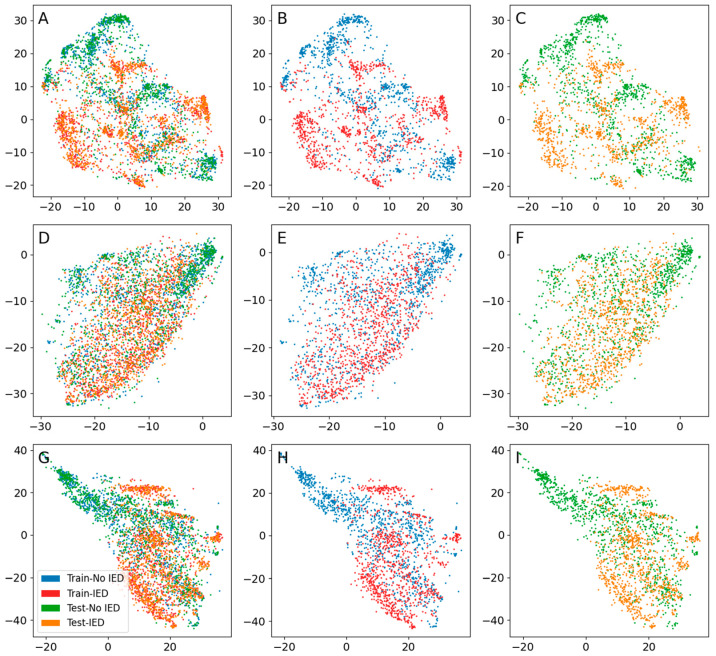
Low-dimensional representations of the IED dataset. Each dot represents 1 s of data. First row (**A**–**C**): CNN. Second row (**D**–**F**): STFT. Third row (**G**–**I**): CWT. First column (**A**,**D**,**G**): all data. Second column (**B**,**E**,**H**): training data. Third column (**C**,**F**,**I**): test data.

The CNN encoder produced the highest clustering score for both the training and test data (Table 3), and there were many visually distinct identifiable clusters (Figure 5B,C). Visually, there was some substructure for the STFT (Figure 5E,F) and CWT encoders (Figure 5H,I), but with a tendency for more confluent patterns and mixing of the categories, and the clustering scores were low.

### 3.3. Seizure Activity

Both the CNN and STFT encoders scored 0 when using a linear kernel (Table 4). For the RBF kernel, the kappa scores were relatively high in general. The CNN encoder scored significantly higher in training using an RBF kernel. There was no significant difference in the performance of the methods applied to the test data using an RBF kernel.

**Figure 6:**
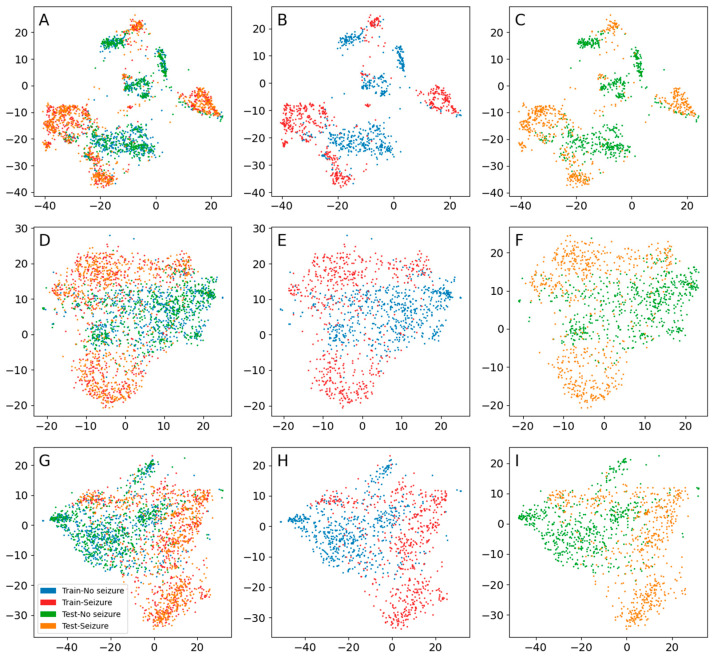
Low-dimensional representations of the seizure dataset. Each dot represents 1 s of data. First row (**A**–**C**): CNN. Second row (**D**–**F**): STFT. Third row (**G**–**I**): CWT. First column (**A**,**D**,**G**): all data. Second column (**B**,**E**,**H**): training data. Third column (**C**,**F**,**I**): test data.

Both with respect to the clustering score (Table 4) and the visual appearance (Figure 6), the CNN encoder performed better for this dataset for both the training and test data. The STFT and CWT encoders performed equally well. Visually, there were substructures, but the clusters were not distinct enough to produce high clustering scores.

## 4. Discussion

In this work on EEG analysis, a novel deep learning approach to t-SNE was compared to conventional parametric t-SNE. Ordinarily, the high-dimensional representation is a preprocessed version of the EEG data. In the presented approach, CNN encoders instead learned a new high-dimensional representation using the principle of t-SNE. Two different time–frequency methods were used for the conventional implementations.

All methods showed promising results. The kappa values calculated using the SVMs were comparable in general, and there was no consistent pattern in favor of any method. On the contrary, the number of clusters as assessed via k-means clustering was consistently higher for the CNN encoders. Distinct clustering is very important in visual analysis. In this study, the data were annotated, and there were thus color codes to guide the eye. However, this may not always be the case, but distinct clusters indicating a closer similarity in the data, i.e., potential categories, are easily identifiable.

All methods can probably be improved further, for example, by optimizing the preprocessing of the data and the hyperparameters of the algorithms. Improvements can also likely be made in a specific sense by tailoring the methods to each specific dataset and categorization problem, e.g., the choice of EEG channels and frequency bands to analyze. There are different ways of implementing time–frequency methods. STFT is a simple linear time-frequency method which provides a cross-terms-free representation, but it has low signal concentration, low resolution, and selectivity [31]. For example, the cross-terms-free Wigner distribution, also known as the S-method, may be a better option, as it also provides a cross-terms-free representation, remedies listed disadvantages of STFT, provides noise influence reduction [32] and shows the best performance in estimation of the instantaneous frequency [33]. Of course, there are many other methods to consider for producing features when using the original parametric t-SNE. However, given the extent of possible variation in deep learning, it is speculated that this approach has the largest potential to improve the performance.

There was a possible tendency for the suggested approach to perform better for IEDs. As IEDs are short transients, it is possible that the convolutional and max-pooling operations were more efficient in detecting these compared to the CWT and STFT representations. The CWT encoder seemed to perform better than the STFT encoder for IEDs. The CWT representation had more dimensions than the STFT representation, but it also consisted of statistical values from the wavelet processing, where the max value was equivalent to the max-pooling operation used in the level analysis of the CNN encoders, and this may have contributed to it performing better for IEDs than the STFT representation.

As evaluated by the SVMs and kappa scores, the methods showed similar performances in the sleep–wake and seizure datasets. These categories are, to a large extent, defined by their frequency content, which is why time–frequency methods can perform well for these data types.

In contrast to the IED and seizure datasets, the number of clusters decreased for the test data compared to the training data of the sleep–wake dataset. The main difference between the datasets, apart from the difference in EEG patterns, was that the test data of the sleep–wake dataset consisted of new subjects, whereas for the other two datasets, the test data consisted of new data from the same subjects that were used for training. This implies overfitting to the training subjects; however, the global structure generalized to new subjects. It is speculated that larger datasets containing data from a larger number of subjects may be necessary for a better generalization to new subjects. Since the CNN encoders learn a new high-dimensional representation, it is reasonable to assume that there is more potential for overfitting in general. This risk is probably higher for smaller datasets, which the demonstration presented in Appendix E indicates, where a smaller dataset was used, and overfitting was more prominent. In the results presented in Section 3, the training data examples are non-overlapping. Using overlapping examples can provide a larger variation in the training data. This is discussed in Appendix A.3, where it is also demonstrated that it can affect the clustering. The effect on overfitting is also demonstrated in Appendix E, where it decreases when using overlapping examples. Other strategies for data augmentation could also be tested. Since the method is self-supervised, pretraining the network on a large unassorted dataset could be a relatively affordable alternative to decrease overfitting. Dropout layers could be added to the model. The model uses L2 regularization for the convolutional and fully connected layers, and other types of regularization could also be tested to mitigate overfitting.

If there is overfitting, it is probably based on the distance between the signals. This is suggested by all of the low-dimensional representations produced using the method in this article, since the resulting clusters conform with the annotations. Hence, the low-dimensional representations of the training data can still be useful and offer insight into the relations between those specific signals, even when the encoders do not generalize well to new data. Whether it is worth the time and resources to train unique encoders on every dataset as a method of analysis is another question.

The suggested approach can be time-consuming to develop. If a high-dimensional representation that produces good results for t-SNE can be identified, then there are faster implementations for the algorithm, e.g., Barnes–Hut t-SNE [34] and Flt-SNE [35], and the deep learning approach may be unnecessarily complicated.

Since the method is self-supervised, anything in the data may induce clustering. This may be beneficial; e.g., the method will, in a sense, be more unbiased. On the other hand, it may also make the method too unselective; e.g., uninteresting artifacts may cause clustering of data that are suboptimal for the task at hand. The method could also be too sensitive; e.g., theoretically, small variations in the electrode placement could affect the generalization if training and test data are recorded on separate occasions, or there may be spurious clustering induced by noise.

The data materials used in this study were relatively small, both with respect to the number of data examples and the number of subjects. Additional materials are needed for further evaluation. Another limitation was that experiments were only performed using the same relatively short example duration. Time–frequency methods may show an inferior performance when using shorter durations. A demonstration using a longer duration is presented in Appendix E, but further testing using different durations is required. The annotation and data selection were performed by one of the authors, and the method was developed by the authors, which must, of course, be evaluated further, using other data and by external researchers.

As described in the introduction, t-SNE has previously been used mainly to visualize EEG data. There are several promising applications: The method could be further developed to produce quick overviews of whole recordings, providing a rapid first assessment evaluating if an EEG is normal or pathological. This could be useful to, e.g., prioritize the order of assessment of EEGs in clinical routine work when the workload is high. The method might also be used to construct trends for long-term monitoring. For example, seizure activity and status epilepticus could easily be detected in intensive care units lacking personnel with training in EEG interpretation. Jing et al. [2] integrated visualization using t-SNE into an annotation tool; rapid annotation of data could be performed by simply manually marking clusters and assigning categories in a graphical user interface, which then automatically annotates the corresponding time intervals in the EEG.

## 5. Conclusions

A novel deep learning approach for t-SNE where CNN encoders learn a new high-dimensional representation was compared to conventional parametric t-SNE using the preprocessed EEG as the high-dimensional representation. The results showed comparable performances regarding the division of the EEG categories, but a superior performance for the CNN encoders in generating more distinctly separated clusters. The data tend to group or cluster in agreement with the traditional EEG categories, which is a first step towards further improvements of EEG categorization. The CNN method shows potential for use in several clinical areas, in addition to research.

## Figures and Tables

**Table 1:** Summary of the main configurations of the encoders of the study.

Encoder	Input Shape	Number of Layers *	Number of Parameters	Number of Latent Dimensions	Learning Rate	Optimizer	Batch Size
CNN	21 × 250	23 + 4 ^†^	19,084 + 1,117,970 ^†^	1260	10^−4^	Adam	500
STFT	630	4	1,823,502	-	10^−4^	Adam	500
CWT	2436	4	2,726,502	-	10^−4^	Adam	500

* Activation and max-pooling layers are not included. ^†^ The first and second numbers refer to the first and second parts of the CNN encoder, respectively.

**Table 2:** Quantitative measures for the sleep–wake dataset. Significantly higher values in a column are in bold (chi-square test); κ indicates the kappa value for SVM; L indicates a linear kernel; R indicates a radial basis function kernel; C indicates the number of clusters by k-means.

	$\kappa_{L}$-Train	$\kappa_{L}$-Test	$\kappa_{R}$-Train	$\kappa_{R}$-Test	C-Train	C-Test
CNN	0.80	0.89	**0.93**	0.86	22	2
STFT	0.77	0.86 *	0.84	**0.92**	2	2
CWT	**0.87**	**0.92**	0.9	**0.92**	4	2

* The significance only applies to the value of the STFT encoder, not the CNN encoder.

**Table 3:** Quantitative measures for the IED dataset. Significantly higher values in a column are in bold (chi-square test). κ indicates the kappa value for SVM; L indicates a linear kernel; R indicates a radial basis function kernel; C indicates the number of clusters by k-means.

	$\kappa_{L}$-Train	$\kappa_{L}$-Test	$\kappa_{R}$-Train	$\kappa_{R}$-Test	C-Train	C-Test
CNN	**0.39**	**0.33**	**0.76**	0.69	18	17
STFT	0	0	0.43	0.35	4	4
CWT	0.31	0.29	0.67	0.56	2	2

**Table 4:** Quantitative measures for the seizure dataset. Significantly higher values in a column are in bold (chi-square test). κ indicates the kappa value for SVM; L indicates a linear kernel; R indicates a radial basis function kernel; C indicates the number of clusters by k-means.

	$\kappa_{L}$-Train	$\kappa_{L}$-Test	$\kappa_{R}$-Train	$\kappa_{R}$-Test	C-Train	C-Test
CNN	0	0	**0.87**	0.8	7	8
STFT	0	0	0.79	0.76	3	3
CWT	**0.66**	**0.68**	0.8	0.74	3	3

**Figure A1:**
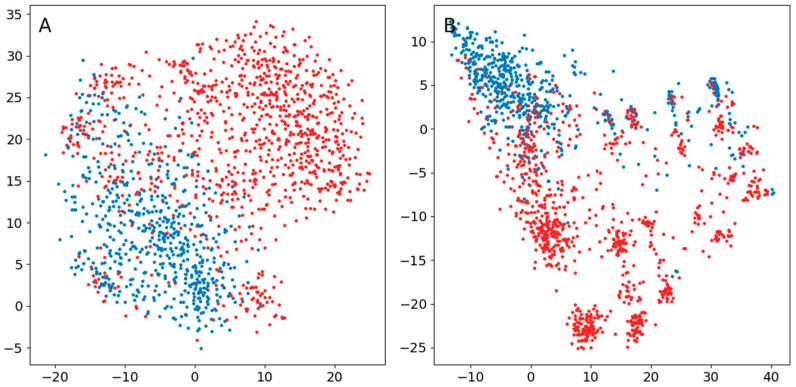
Comparison of training encoders using non-overlapping or overlapping time intervals as examples. EEG with a 1601 s duration representing status epilepticus, where each dot represents 1s of data and red dots indicate seizure activity. (**A**) Non-overlapping examples. The EEG was divided into 1601 unique examples by using consecutive 1 s intervals. (**B**) Overlapping examples. The whole EEG recording was loaded into CPU memory, and examples were then generated online during training by randomly selecting time positions in the recording and extracting 1 s of data.

**Figure A2:**
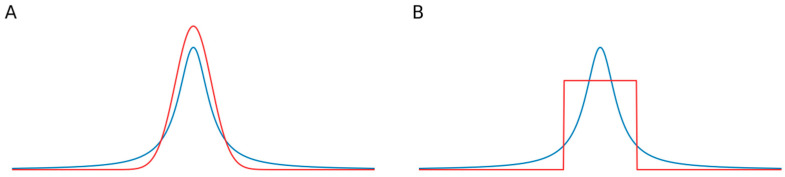
Theoretical probability distributions. The blue curve is the t-distribution with one degree of freedom. (**A**) The normal distribution is in red. (**B**) The suggested distribution is in red.

**Figure A3:**
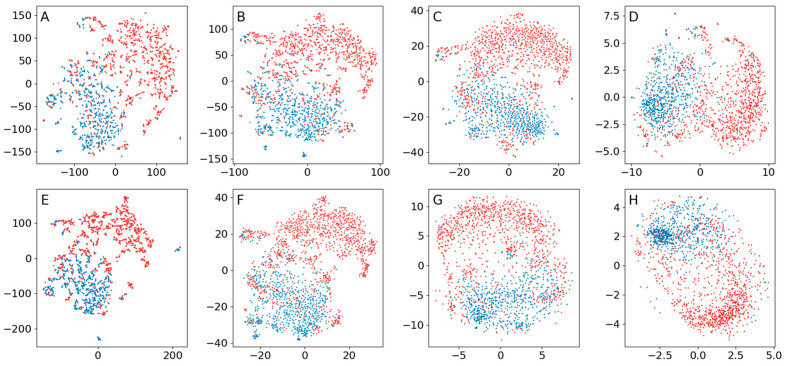
Comparison of the original t-SNE performed with the suggested distribution or a normal distribution. The data are short-time Fourier transform representations of an EEG representing status epilepticus, where each dot is 1 s and red dots indicate seizure activity. First row (**A**–**D**): using a normal distribution. Second row (**E**–**H**): using the suggested distribution. (**A**) Perplexity 0.25%; (**B**) perplexity 1%; (**C**) perplexity 5%; (**D**) perplexity 20%; (**E**) 0.25% neighbors; (**F**) 1% neighbors; (**G**) 5% neighbors; (**H**) 20% neighbors.

**Figure A4:**
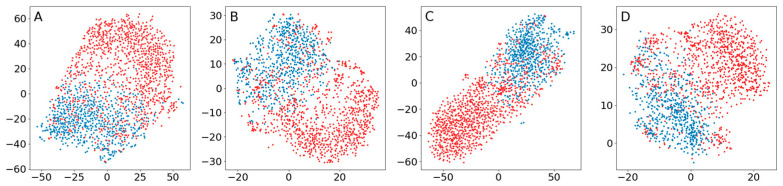
Comparison of parametric t-SNE and CNN encoders when using the normal and suggested distributions. Batch size during training with a batch size of 500 and perplexity/number of neighbors of 5. (**A**) Parametric t-SNE with a normal distribution and STFT features. (**B**) Parametric t-SNE with the suggested distribution and STFT features. (**C**) CNN encoder with a normal distribution. (**D**) CNN encoder with the suggested distribution.

**Figure A5:**
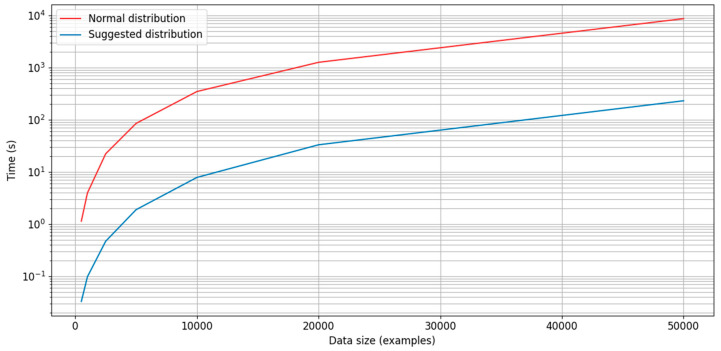
Comparison of processing times for estimating the normal distribution (red) and the suggested distribution (blue) in the present work. Estimations are 34–47 times faster when using the suggested distribution. However, in practice, the estimation only constitutes part of the computations during training; training times depend on the batch size, and for a batch size of 500, training times were approximately 2–2.5 times faster when using the suggested distribution.

**Figure A6:**
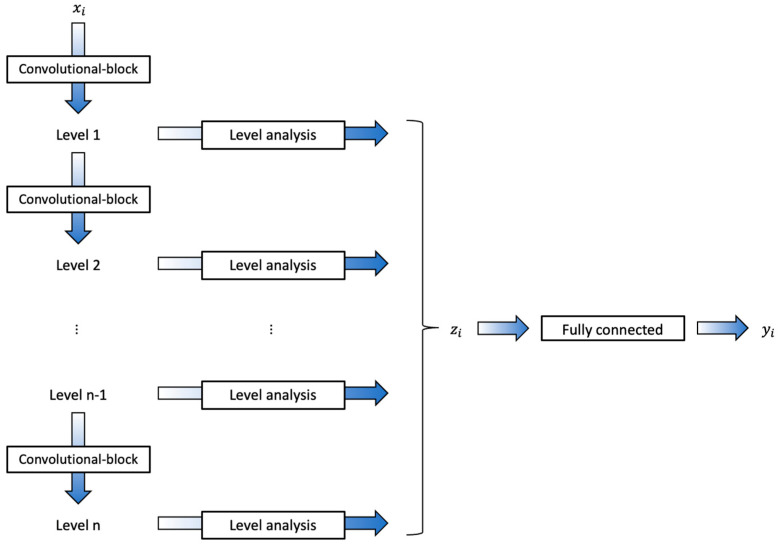
Overview of the encoder architecture. To the left, a series of convolutional blocks process and successively down-sample the data $x_{i}$ through max-pooling to produce levels of an increasing field of view. Each resulting level is analyzed further, and the results from all levels are concatenated to produce a latent representation $z_{i}$ of the data $x_{i}$. A series of fully connected layers then produce the low-dimensional representation $y_{i}$.

**Figure A7:**
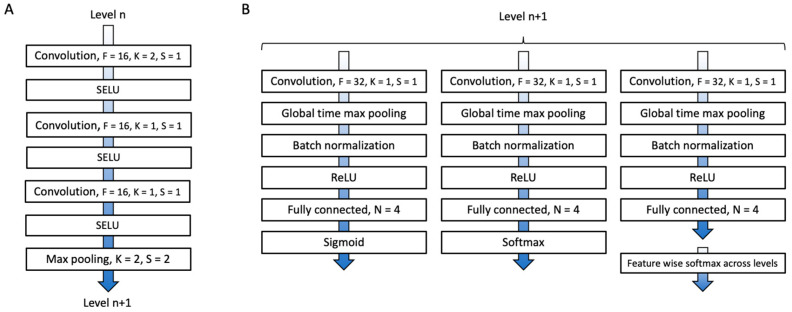
(**A**) Illustration of a convolutional block. (**B**) Illustration of level analysis. F: number of filters; K: kernel size; S: strides; N: number of nodes; ReLU: rectifying linear unit.

**Figure A8:**
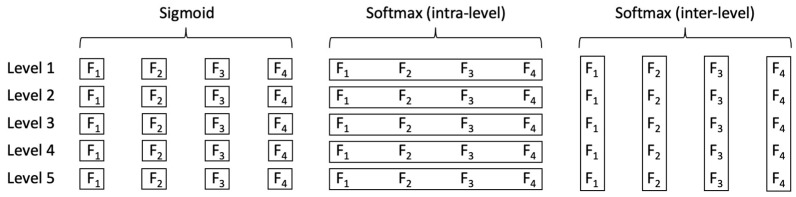
Illustration of the activations for the latent representation of five levels. Boxes indicate dependence; for the sigmoid activations, each feature F_i_ can take any value between 0 and 1, independent of other features Fj, i≠j; for softmax activations, the values of the features in each box are normalized to 1 so that $\overset{4}{\underset{i=1}{\sum }}F_{i}=1$.

**Figure A9:**
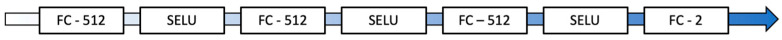
Illustration of the fully connected block mapping $z_{i}$ to $y_{i}$. FC: fully connected layer with the number of nodes indicated.

**Figure A10:**
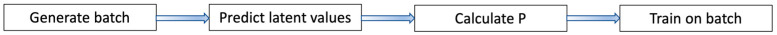
Illustration of the pipeline for generating a training batch. First, examples x are randomly selected. The latent values are then predicted using the first part of the encoder. Thereafter, the probability distribution P is calculated, and x and P are then fed into the full encoder for training.

**Figure A11:**
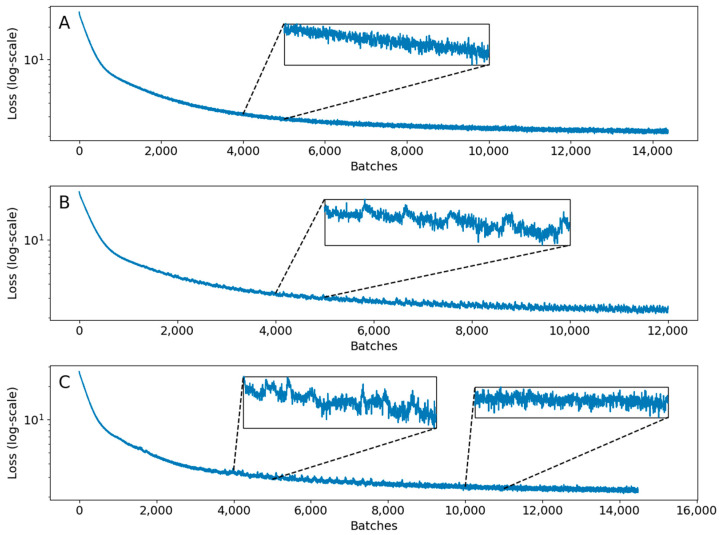
Examples of training losses. (**A**) Example where the loss decreases steadily apart from the smaller fluctuation in the random character. (**B**) Example where the loss shows instability in the form of a low-amplitude sawtooth pattern which represents movement between local minima with different topologies for the low-dimensional representation. (**C**) Example where the loss shows transient instability but then stabilizes and converges to one topology.

**Figure A12:**
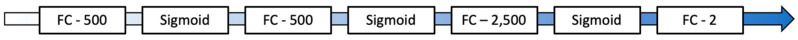
Illustration of the encoder of parametric t-SNE. FC: fully connected layer with the number of nodes indicated; Sigmoid: sigmoid activation.

**Figure A13:**
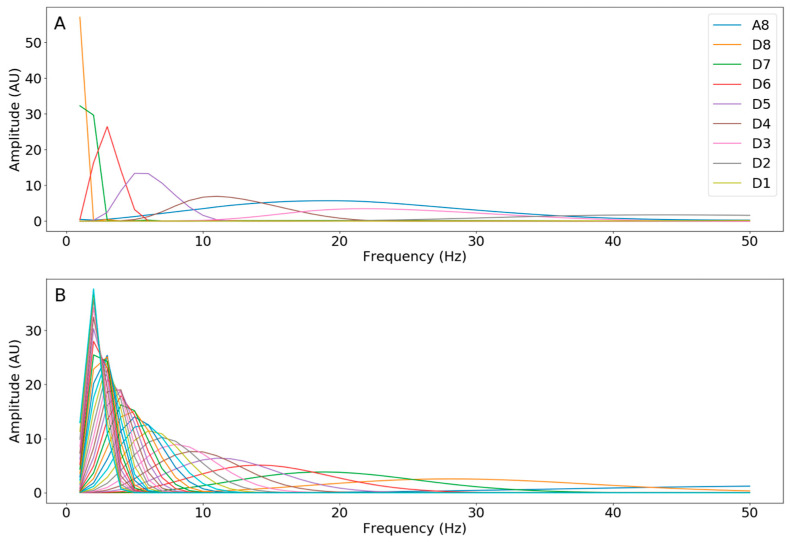
Frequency response for 1, 2, 3, …, 50 Hz. (**A**) The Daubechies wavelet (db4) with eight levels. (**B**) The Ricker wavelet with widths of 1, 2, 3, …, 30, where the maximum of each corresponding band has a frequency that decreases with increasing width.

**Figure A14:**
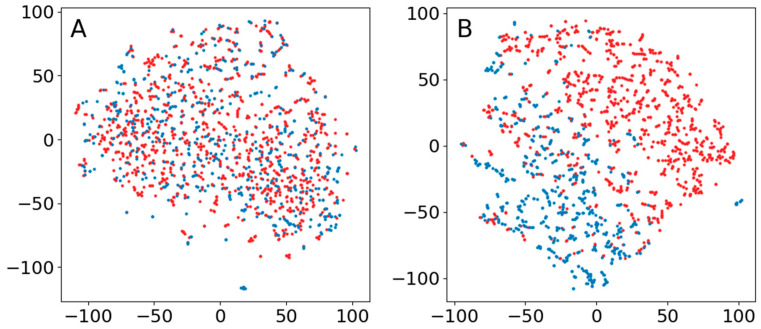
Results of t-SNE with perplexity 0.25% for an EEG representing status epilepticus. Each dot represents 1 s and red dots indicate seizure activity. (**A**) The Daubechies wavelet (db4) using six levels. (**B**) The Ricker wavelet with widths of 2, 3, 4, …, 30.

**Figure A15:**
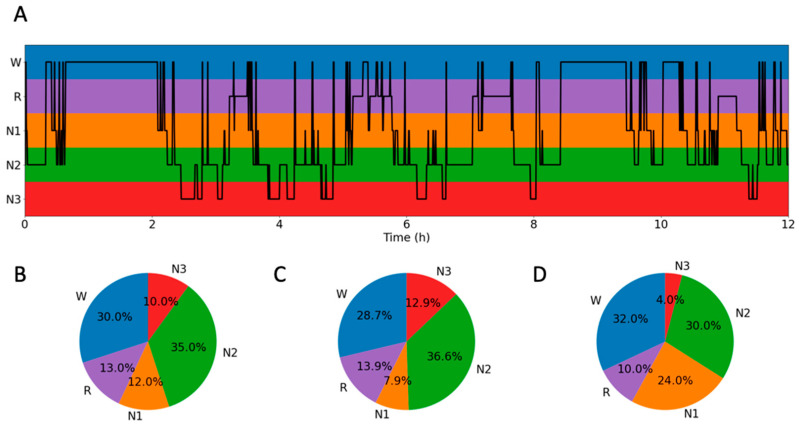
Sleep stages of a 12 h recording. W (blue): wakefulness; R (purple): REM sleep; N1 (orange): non-REM sleep stage 1; N2 (green): non-REM sleep stage 2; N3 (red): non-REM sleep stage 3. (**A**) Overview of the sleep stages. (**B**) Distribution of the sleep stages for the whole recording. (**C**) Distribution of sleep stages for training data. (**D**) Distribution of sleep stages for test data.

**Figure A16:**
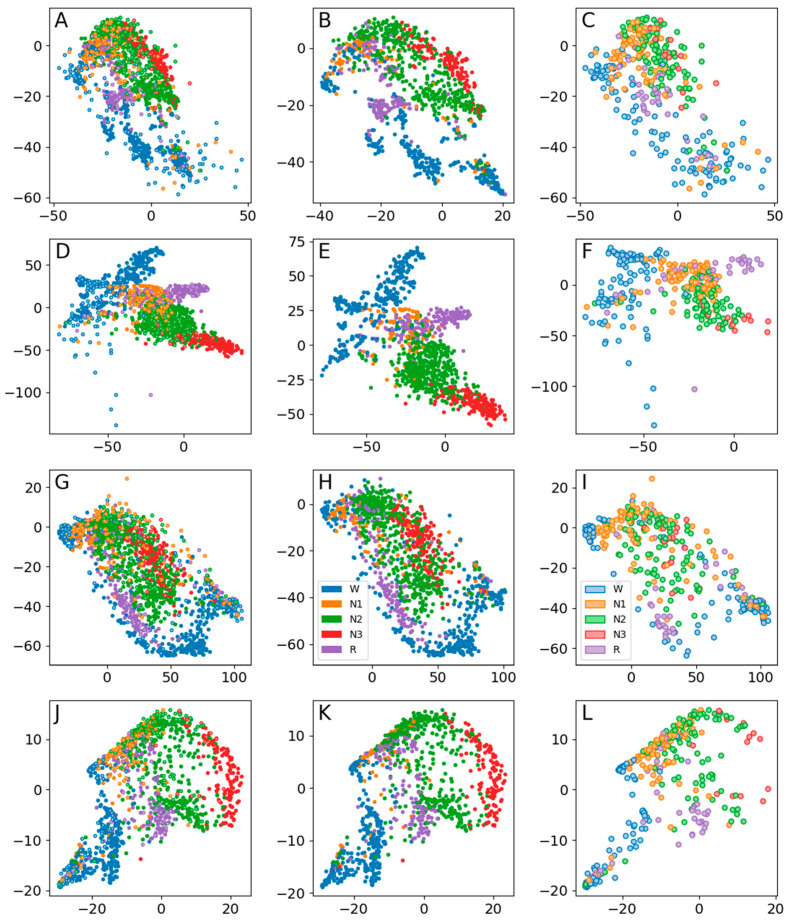
Low-dimensional representations of the 12-h dataset. Each dot represents 30 s of data. Blue = awake; orange = sleep stage N1; green: sleep stage N2; red: sleep stage N3; purple: REM. First row (**A**–**C**): CNN encoder. Second row (**D**–**F**): STFT encoder. Third row (**G**–**I**): CWT encoder. Fourth row (**J**–**L**): CNN encoder using overlapping examples. First column (**A**,**D**,**G**,**J**): all data. Second column (**B**,**E**,**H**,**K**): training data. Third column (**C**,**F**,**I**,**L**): test data.

## Data Availability

The data used in this study were obtained from the Temple University Hospital EEG data corpus (https://isip.piconepress.com/projects/tuh_eeg/; accessed on 17–21 January 2019).

## Appendix A

### Appendix A.1. Data Selection and Annotation

Three datasets for clinically significant EEG activities were created to test the proposed method: (1) sleep vs. wakefulness; (2) IEDs vs. no IEDs; and (3) seizure activity vs. seizure-free activity.

To produce the project’s database, a set of 1000 EEGs from 1000 unique subjects were first extracted from the TUH EEG Corpus [22] via an automated search. Inclusion criteria were as follows: subject age ≥18 years, sampling rate 250 Hz, and duration of at least 600 s. This resulted in a set of subjects with an average age of 51 years (553 females) and recordings with an average duration of 1356 s. From this database, a subset of EEGs containing (1) wakefulness, (2) sleep, (3) frequent IEDs, and (4) seizure activity was selected. The number of EEGs was limited by their natural occurrence in the extracted material. For category 2, the EEGs were dominated by sleep, but it was difficult to find complete EEGs without some amount of wakefulness. These EEGs were therefore dichotomously annotated as 10 s epochs so that each epoch represented wakefulness or sleep. Sleep was defined according to the American Academy of Sleep Medicine’s (AASM) sleep scoring manual [36] but modified to 10 s instead of 30 s, and sleep stages were not indicated. IEDs were annotated according to the criteria proposed by Kane et al. [37]. Seizure activity was defined according to Hirsch et al. [38]. Selection and annotation were performed by a specialist in clinical neurophysiology (author M.S.).

The above procedure resulted in 19 EEGs of wakefulness, 14 EEGs of dominating sleep, 31 EEGs with frequent epileptiform activity, and 6 EEGs containing seizure activity. From these EEGs, the datasets were created using examples with a 1 s duration, and the sets where split with probabilities of 0.75 for training and 0.25 for testing. The sleep–wake dataset had the largest number of subjects and examples. This dataset was split with respect to subjects, i.e., testing would evaluate generalization to new subjects, which resulted in 14 awake and 5 sleeping subjects for training and 8 awake and 3 sleeping subjects for testing. The IED and seizure datasets were split with respect to examples, i.e., testing would evaluate generalization to new data from the same subjects. A summary of the number of examples per dataset and the respective categories of the datasets is presented in Table A1.

**Table A1:** Summary of the number of examples per dataset and category using examples with a 1 s duration. Sleep–wake: 0 = awake, 1 = sleep. IEDs: 0 = no IED, 1 = IED. Seizure: 0 = no seizure activity, 1 = seizure activity.

	Train 0	Train 1	Test 0	Test 1
Sleep–wake	17,386	10,990	7261	3460
IEDs	5932	3246	2012	1138
Seizures	5592	1949	1875	600

These datasets had not been used in the previous development of the method. For this, a few of the 1000 extracted EEGs, not used as training or test data for the results, were used for preliminary testing of the Python scripts before the final analysis using the three datasets. In particular, one longer recording was utilized, and it was also used for the demonstration in Appendix E. One of the seizure EEGs, representing status epilepticus, was reused here in the appendices to generate the illustrative examples.

### Appendix A.2. Example Duration

To be able to identify patterns, it is necessary to have a sufficient number of data points, as is the case when training deep learning models. For a standard 20-min recording, an example duration of 1s returns 1200 unique examples, compared to, e.g., 40 unique examples when using an example duration of 30 s. For instance, if an EEG contains one 15 s episode of seizure activity, an example duration of 1 s will return 15 examples, whereas a longer duration may only return a few examples, or the activity may only constitute part of the duration of an example. For EEGs with frequent IEDs, a longer duration could result in almost all examples containing IEDs, making it hard to study this waveform. Most waveforms of interest will fit within 1 s, but some patterns, e.g., seizure activity and sleep stages, may become poorly represented. For sleep stages, during an annotated epoch, the character of the activity can change, meaning that different parts of the epoch could represent different sleep stages. Thus, using 1 s examples with data annotated for 10 or 30 s epochs probably results in some examples being mislabeled. However, annotating sleep for 1 s epochs would be very time-consuming, and probably produce a large scoring variability since there is no clear definition for sleep staging of such short time intervals. The chosen duration of 1 s was thus a compromise, mainly to allow for a larger number of examples for training and testing. For the patterns with a long duration, some degree of label noise was expected. A demonstration of the use of a longer duration is presented in Appendix E.

### Appendix A.3. Overlapping vs. Non-Overlapping Examples

A way to counteract overfitting in machine learning is to use overlapping examples, thereby increasing the variation in the training data. Shorter signal transients, e.g., IEDs, may appear at any time position in an EEG. Therefore, using a limited amount of non-overlapping examples may result in encoders only detecting transients at certain positions in an example. The effect on the result of using overlapping vs. non-overlapping examples may sometimes be pronounced (Figure A1). The effect on generalization is demonstrated in Appendix E.

As described below (D.2), time–frequency methods were used for comparison in the evaluation of the method. Here, using overlapping examples, the amount of data can expand substantially. As an example, a 21-channel 20 min EEG recording with a sampling frequency of 250 Hz has 6,300,000 data points. If all data are used to create non-overlapping examples, then this will return 1200 unique examples. The total data size will be the same if the whole recording is used to generate random overlapping examples online during training. A time–frequency method that represents each example using 630 values will reduce the amount of data when using non-overlapping unique examples (756,000 data points), but increase it when using overlapping examples and retaining the time resolution (189,000,000 data points). Using randomly selected overlapping examples therefore proved hard to implement for the time–frequency methods. In the present study, fixed non-overlapping examples were used in all instances to make the comparisons fairer. This limitation of the study was thus due to the time–frequency methods utilized.

## Appendix B

### Appendix B.1. The Original t-SNE

In the original t-SNE developed by van der Maaten and Hinton [1], the joint probability $p_{ij}$ of a pair of high-dimensional data points $x_{i}$ and $x_{j}$ being neighbors is assumed to follow a normal distribution according to

(A1) $$p_{ij}=\frac{exp\left(-\|x_{i}-x_{j}\|{}^{2}/2\sigma_{i}^{2}\right)}{\sum_{k\neq l}exp\left(-\|x_{k}-x_{l}\|{}^{2}/2\sigma_{i}^{2}\right)},$$

where $\left\|x_{i}-x_{j}\right\|$ is the Euclidean distance $d_{ij}$, and $\sigma_{i}$ is the standard deviation. $\sigma_{i}$ is determined by a binary iterative search to meet a condition imposed by the perplexity parameter. The perplexity is $2^{H\left(P_{i}\right)}$, where $P_{i}$ is the probability distribution corresponding to $\sigma_{i}$, and $H\left(P_{i}\right)=-\underset{j}{\sum }p_{j|i}\log_{2}p_{j|i}$ is the Shannon entropy. Thus, the perplexity determines $\sigma_{i}$ and reflects the number of efficient neighbors. In the original implementation, the diagonal is set to zero ($p_{ij}=0,\;i=j$), and the symmetrical matrix $p_{ij}=p_{i|j}+p_{j|i}=p_{i|j}+p_{i|j}^{T}$ is used.

For calculating the probability $q_{ij}$ of a corresponding pair of low-dimensional data points $y_{i}$ and $y_{j}$ being neighbors, a t-distribution was introduced by van der Maaten and Hinton [1]:

(A2) $$q_{ij}=\frac{{\left(1+\|y_{i}-y_{j}\|{}^{2}\right)}^{-1}}{\sum_{k\neq l}{\left(1+\|y_{k}-y_{l}\|{}^{2}\right)}^{-1}}$$

To match the resulting probability distributions P and Q, the Kullback–Leibler divergence

(A3) $$KL\left(P||Q\right)=\sum_{i}\sum_{j}p_{ij}log\frac{p_{ij}}{q_{ij}},$$

which reflects the difference between the distributions of the probabilities, is used as the loss function. Gradient descent is used to minimize the loss function.

### Appendix B.2. Modified t-SNE Using Custom Probability Distributions

The difference between the normal distribution and the t-distribution, i.e., that the t-distribution has heavier tails, is supposed to create favorable attractions and repulsions, resulting in preservation of the local structure while overcoming the crowding effect [1] (Figure A2A). In the suggested approach of the present work, the high-dimensional representation was generated by the encoder. The representation thus changed during training and the corresponding probabilities had to be recalculated for each training batch. The original t-SNE uses an iterative search to determine the standard deviation of the normal distribution given the perplexity. This can be time-consuming (see Appendix B.5); therefore, a custom distribution based on ranked distances was used instead.

By ranking the distances $d_{ij}$ between examples, custom distributions based on the rank $r_{ij}$ can be constructed. A simple alternative is to assume that each example has a certain number n≥1 of close neighbors, and that all other examples are distant. Thus, by setting the n lowest ranked distances $d_{ij}$ to a value of a and the rest to a value of b≪a, and then normalizing with the sum c of all values, this results in a probability distribution with probabilities (Figure A2B).

(A4) $$p_{ij}=\left\{\begin{array}{c} \frac{a}{c}\;if\;r_{ij}\leq n\\ \frac{b}{c}\;if\;r_{ij}>n \end{array}\right.$$

If the number of examples is N, then $c=N\left(an+b\left(N-n\right)\right)$. In this approach, the information regarding distances and ranks is lost.

### Appendix B.3. Comparison of the Use of a Normal Distribution or a Distribution Based on Ranked Distances in the Original t-SNE

To illustrate the performance using the suggested distribution, the original t-SNE was performed using either its original implementation with a normal distribution or a modified version with the suggested distribution. The code of van der Maaten for a Python implementation of t-SNE was used (https://lvdmaaten.github.io/tsne/code/tsne_python.zip; accessed on 25 August 2021) to perform the tests. A modified version was created for the suggested distribution. The original code has a default value of 50 components for PCA, which was used. The low-dimensional representation $y_{i}$ was initialized randomly from a uniform distribution, and the same randomization was used in all examples. Early exaggeration was used with a factor of 12 for the first 250 iterations, as suggested by Kobak and Berens [39], and 5000 iterations were performed. t-SNE was performed for an EEG using a time–frequency representation (STFT; Appendix D.2.1) for perplexities of 0.25, 1, 5, and 20 percent.

The resulting low-dimensional representations were similar (Figure A3). For higher perplexity values, the suggested distribution seemed to produce less structure, i.e., more confluent patterns. However, it is possible that the perplexity and the number of neighbors n are not equivalent. The range of values for the low-dimensional representations differ, where the range decreases more rapidly with an increasing number of neighbors for the suggested distribution compared to the normal distribution.

### Appendix B.4. Comparison of the Use of a Normal Distribution or a Distribution Based on Ranked Distances When Training Encoders

To illustrate the effects of the use of a normal distribution or the suggested distribution when training encoders on performance, parametric t-SNE encoders (Appendix D.2.) and CNN encoders (Appendix C) were trained using either of these distributions. A batch size of 500 was used, and the perplexity and number of neighbors n were both set to 5. All resulting low-dimensional representations showed a strong division according to the categories, but possibly a tendency for a more local structure when using the suggested distribution (Figure A4).

Generally, the best results were produced using low values for the number of neighbors n (0.5–2 percent of the batch size) (Figure A3). In the article, n=5 was used, which, for a batch size of 500, is equal to one percent. This is in line with the rule of thumb for the value of perplexity suggested by Kobak and Berens [39].

### Appendix B.5. Time Consumption for a Normal Distribution and a Distribution Based on Ranked Distances

The algorithms for estimating the normal and suggested distributions were performed on examples having 50 components and batches of 500, 1000, 2500, 5000, 10,000, 20,000, and 50,000 examples. The tests were performed on a computer with an Intel Core i7-6800K CPU 3.4 GHz (6 cores/12 threads).

In computing the suggested distribution, the distance involves matrix multiplication ($O(n^{3})$), and the sorting should take O(nlogn) on average, but in the worst case, it may take $O(n^{2}$) (https://numpy.org/doc/stable/reference/generated/numpy.sort.html accessed on 25 August 2021), and there are n instances of sorting. In the tests, the processing times using either distribution followed the same time complexity, but the use of a normal distribution consumed more time by two orders of magnitude(Figure A5). However, since computing the distributions only constitutes part of all computations during training, for a batch size of 500, training times were 2–2.5 times faster when using the suggested distribution.

## Appendix C

### Appendix C.1. Encoder Architecture

In all encoders, the input $x_{i}$ was processed through a series of blocks of convolutions and max-pooling, with the intention of producing levels with an increasing field of view of the input $x_{i}$ (Figure A6). Each level was then analyzed through further convolution and max-pooling, followed by fully connected layers, to generate a set of values characterizing it. The values from all levels were concatenated and became the latent representation $z_{i}$. Each EEG channel was analyzed separately throughout the convolutional blocks and level analysis. A fully connected block of four layers then mapped $z_{i}$ to a two-dimensional representation $y_{i}$. To be able to access the latent representation, all types of encoders were implemented in two parts: from $x_{i}$ to $z_{i}$, and from $z_{i}$ to $y_{i}$. Arranging the two parts in series produced the full encoder.

#### Appendix C.1.1. Convolutional Blocks

The convolutional blocks (Figure A7A) consisted of 3 convolutional layers, each with 16 filters and strides of 1. The first layer had a kernel size of 2, while the second and third layers had kernel sizes of 1. Scaled exponential linear units (SELUs) [40], which have self-normalizing properties, were used as activations between the layers. Each block was finished with max-pooling with a kernel size of 2 and strides of 2, thus down-sampling by a factor of 2.

#### Appendix C.1.2. Level Analysis

One aspect of the idea of the encoder architecture is that the resulting values from the level analysis represent the presence of high-level waveform features in different fields of view. It was assumed that some of these could be independent features, but there could also be dependent features, either being mutually exclusive or of varying importance. Dependence could be found either for a certain level or across different levels. For example, from the EEG experts’ perspective, there may be artifacts but, at the same time, visible cortical activity; activity can be classified as either regular or irregular; and waveforms with a pointy morphology may be of greater importance than those with a round morphology.

As an implementation of this idea, the level analysis consisted of three parallel blocks (Figure A7B), each starting with a convolutional layer (32 filters, a kernel size of 1, and strides of 1), followed by max-pooling across the time dimension, reducing this dimension to 1, and then batch normalization, a rectifying linear unit activation, and a fully connected layer (four nodes). The first block ended in a sigmoid activation, constituting independent features (Figure A8, left). The second ended in a softmax activation, constituting intra-level dependent features (Figure A8, middle). In the third block of all levels, groups were formed consisting of one feature from each level, and each group was put through a softmax activation, constituting inter-level dependent features (Figure A8, right).

For down-sampling by a factor of 2 and using the sampling frequency of 250 Hz from the original data, the timestep of the first and second levels would correspond to 8 ms (125 Hz) and 16 ms (62.5 Hz), respectively. Since the idea was to create an encoder that mimics how an expert visually analyzes an EEG, the data were low-pass filtered at 40 Hz (which is traditionally used by most clinical neurophysiologists at the authors’ clinic), the first two levels were skipped, and the remaining five levels (32, 64, 128, 256, and 512 ms) were used. This resulted in a latent representation of 1260 dimensions.

#### Appendix C.1.3. Fully Connected Block

The mapping of $z_{i}$ to $y_{i}$ was accomplished using three fully connected layers, each with 512 nodes, followed by a final fully connected layer with two nodes (Figure A9). SELU activations were used between the fully connected layers.

### Appendix C.2. Loss Function

Before training on a batch, the high-dimensional representation had to be predicted by the first part of the encoder (Figure A10). From this, the probability distribution P could be computed.

The loss function thus consisted of calculating the resulting probability distribution Q of the low-dimensional representation y, given by (B.2), and then the Kullback–Leibler divergence of P and Q, given by (B.3).

### Appendix C.3. Hyperparameters

The method described in this paper was developed over a long period of time using various data. The encoder architecture slowly evolved and emerged as an alternative that, on average, produced consistent and interesting results. When training and using validation data, at least for small datasets, there was usually some degree of overfitting to the training data. However, it was almost always the case that if the training loss converged, then the validation loss would also converge. Given this previous experience, in the present evaluation, no validation data were used during training. Only the training loss was monitored, and the only parameter considered for adjustment was the learning rate.

The number of filters was limited by memory constraints; extraordinarily small kernels were chosen to reduce the memory load. The number of nodes of the fully connected layers of the level analysis was selected to offer a high-dimensional representation with a size in the same range as the comparative methods (630–2436 dimensions; Appendix D.2.1 and Appendix D.2.2). The number of nodes of the fully connected layers of the mapping from $z_{i}$ to $y_{i}$ was selected to obtain a total number of parameters that was less than that for the parametric t-SNE encoders (D.1). These last two choices were made to conduct fairer comparisons with parametric t-SNE. The first part of the encoder had 19,084 parameters (18,124 trainable) and the second part had 1,171,970 parameters (all trainable).

Adam was used as the optimization algorithm [41] with parameters β1 = 0.5 and β2 = 0.9. All convolutional and fully connected layers used L2 kernel regularization (default value of 0.01), and all kernels of these layers that were followed by SELU activations were initialized using the LeCun normal distribution as recommended in the TensorFlow documentation (https://www.tensorflow.org/api_docs/python/tf/keras/activations/selu 25 August 2021). A batch size of 500 was used.

A learning rate of 10^−4^ was used for the data utilized to produce the results, and all examples of low-dimensional representations, except for the sleep scoring data when training with overlapping examples in Appendix E, where 5 × 10^−6^ was used. The loss will show less variation in the random character (Figure A11A), which is mostly due to variation in the data content from batch to batch. If there are fluctuations in the loss’s baseline with local trends, then the encoder is probably moving between different local minima and the training may not satisfactorily converge (Figure A11B); if the low-dimensional representation is visualized intermittently during training, then it may show a dramatically changing topology. However, the training may still stabilize, producing a satisfactory result (Figure A11C).

A general recommendation would be to start the training with a learning rate of 10^−4^. If the loss does not decrease steadily and converge, then the training can be restarted using a lower learning rate. Restarting several times using incrementally lower rates (5 × 10^−5^, 1 × 10^−5^, 5 × 10^−6^, etc.) may be necessary.

Training was performed until the loss stopped decreasing. This was decided by comparing the average loss of the last 2500 iterations with the preceding 2500 iterations to a precision of 2 decimals. The optimal length of the intervals over which the averages are calculated that works best depends on the data and the learning rate. Therefore, the intervals may have to be increased when using a lower learning rate to prevent premature stopping, or decreased for a specific dataset to prevent the training from continuing after the loss has converged satisfactorily. The resulting training times for the three datasets were 12–13 h each when using a single GPU.

## Appendix D

### Appendix D.1. Parametric t-SNE

The original implementation of parametric t-SNE [14], also used by Li et al. [15] and Xu et al. [16], utilizes a network consisting of a series of fully connected nodes with sigmoid activations between them. Xu et al. [16] used three fully connected layers, with 500, 500, and 2500 nodes, respectively, followed by a final fully connected layer with d nodes (Figure A12). The latter architecture was used in the implementation of the present study with d=2.

In the original implementation, greedy layer-wise training is used to pretrain the encoders, followed by fine-tuning using t-SNE. In the present study, the encoders were trained directly using the principle of t-SNE. The same parameters as for the CNN encoders were used (batch size of 500, learning rate of 10^−4^, and the same stopping criteria).

Two different types of feature representations (STFTs, Appendix D.2.1; CWTs, Appendix D.2.2) were used, and the corresponding encoders had 1,823,502 and 2,726,502 parameters (all trainable).

### Appendix D.2. Features

In this work, two different time–frequency methods were used to generate features for parametric t-SNE: short-time Fourier transforms (STFTs) and continuous wavelet transforms (CWTs). When producing transforms of the datasets described in Appendix A, the same randomizations were used so that all examples represented the same time interval across the different data representations and datasets.

#### Appendix D.2.1. STFTs

STFTs produce time series based on Fourier transforms for fixed sized windowed signals [31]. All data were transformed using the STFT implementation in the SciPy library of Python (scipy.signal.stft), where each timestep represented 1 s. The default Hann window was used. The absolute amplitude values were taken. Frequencies up to 30 Hz (~0.98–29.3 Hz) were used, and the total number of values was 630 per example (21 channels, 30 bands). The transform was first applied to whole continuous recordings before examples were extracted.

#### Appendix D.2.2. CWTs

In their work using parametric t-SNE, Li et al. [15] and Xu et al. [16] used the Daubechies wavelet [24]. This is a discrete wavelet for which the resulting frequency bands, usually referred to as levels, have different time resolutions. This has its benefits but can also make them harder to use, and the duration of the examples will limit the frequency resolution. The frequency bands are relatively wide and sparse (Figure A13A); for some applications, this may be a useful property due to the removal of unnecessary information and compression of the data. However, for other applications, it may be detrimental; e.g., seizure activity may have a frequency evolution [38], and, at least in theory, if the frequency bands are too wide and sparse, then this may not be detected efficiently.

Since most signal examples in this study had a short duration, the continuous Ricker wavelet (also referred to as the Marr or Mexican hat wavelet) [25] was also considered. The frequency bands can be made denser, thus providing a better frequency resolution (Figure A13B), but this comes at the expense of a greater amount of overlapping among the bands. Tests were performed using the Python libraries pywt [42] for the Daubechies wavelet and scipy.signal.cwt for the Ricker wavelet. For examples with a 1 s duration, the Ricker wavelet showed a superior performance over the Daubechies wavelet when applying the original t-SNE to the resulting data (Figure A14A,B).

Based on the above, the Ricker wavelet was thus chosen for this study, using widths of 2, 3, 4, …, 30. The transform was applied to whole continuous recordings before extracting examples. For each channel and wavelet band of an example, the mean, standard deviation, minimum value, and maximum value were calculated, producing 2436 values in total per example.

## Appendix E

### Appendix E.1. Data

All but one of the extracted EEGs (1000) were standard exams that took about 20 min. The exception was a 12 h recording from one subject containing sleep and wakefulness with a full range of sleep stages. This EEG was used for some tests during the development of the method. The results presented here should therefore not be regarded as an evaluation test, but rather a demonstration of the method using examples with a longer duration. It has therefore been placed in the appendix.

The EEG was divided into 30 s epochs and scored according to the AASM sleep scoring manual. An overview of the resulting distribution of sleep stages is presented in Figure A15A,B. The 30 s duration was retained, resulting in 1440 examples in total. The first 1080 examples were used for training, and the remaining 360 examples were used for testing. Overviews of the distribution of sleep stages for the training and test data are presented in Figure A15C,D.

### Appendix E.2. Training Encoders

Training was performed using a learning rate of 10^−4^ and a batch size of 500, and the number of neighbors and perplexity were set to 5. For the CNN encoders, due to memory constraints, to permit the use of the same batch size, the data were preprocessed: They were low-pass-filtered at 30 Hz and then down-sampled to 62.5 Hz. It was also necessary to reduce the number of filters in the convolutional layers to half of what had been previously used. The time–frequency representations for the parametric t-SNE were generated as previously described (Appendix D). However, for the STFT features, for each channel and frequency band, the average values were calculated for the whole 30 s duration.

In addition, training was also performed for a CNN encoder using overlapping examples. A lower learning rate of 5 × 10^−5^ was necessary for stable training. The training time for non-overlapping examples was approximately 20 h, while for overlapping examples and the lower learning rate, it was approximately 2 days and 14 h.

### Appendix E.3. Results

Training with non-overlapping examples resulted in similar kappa scores (Table A2), and the STFT encoder produced the highest scores, but there were no significant differences except for when using the training data and linear kernel. In all instances, the test scores were lower than the training scores, indicating overfitting. A probable reason for this more apparent overfit, compared to the result in Section 3 of the article, is that the dataset used here was smaller and there were five categories instead of two. The training clustering score for the CNN encoder was the highest. Possibly due to outliers, the test clustering score for the STFT encoder was moderately high. The other clustering scores were low.

When training with overlapping examples, the results also showed overfitting. However, compared to the previous results for non-overlapping examples, it had decreased, and the test scores were significantly higher. For the linear kernel, the test score increased from 0.35 to 0.53, and for the RBF kernel, it increased from 0.19 to 0.52. This comparison, of course, was not fair to the STFT and CWT encoders, since no evaluations were performed using overlapping examples.

Visually, the results were in line with the quantitative measures (Figure A16). Separation and clustering were better for the STFT and CNN encoders when training with overlapping examples.

**Table A2:** Quantitative measures for the 12 h dataset. Significantly higher values in a column are in bold (chi-square test). κ indicates the kappa value for SVM; L indicates a linear kernel; R indicates a radial basis function kernel; C indicates the number of clusters by k-means.

	$\kappa_{L}$-Train	$\kappa_{L}$-Test	$\kappa_{R}$-Train	$\kappa_{R}$-Test	C-Train	C-Test
CNN	0.55	0.35	0.85	0.19	12	2
STFT	**0.77**	0.51	0.96	0.33	2	6
CWT	0.36	0.30	0.92	0.30	2	2
CNN *	0.63	**0.53**	0.79	**0.52**	3	2

* Training with overlapping examples.
